# PATRISTIC: a program for calculating patristic distances and graphically comparing the components of genetic change

**DOI:** 10.1186/1471-2148-6-1

**Published:** 2006-01-03

**Authors:** Mathieu Fourment, Mark J Gibbs

**Affiliations:** 1 School of Botany and Zoology, The Australian National University, Canberra ACT 0200, Australia

## Abstract

**Background:**

Phylogenies are commonly used to analyse the differences between genes, genomes and species. Patristic distances calculated from tree branch lengths describe the amount of genetic change represented by a tree and are commonly compared with other measures of mutation to investigate the substitutional processes or the goodness of fit of a tree to the raw data. Up until now no universal tool has been available for calculating patristic distances and correlating them with other genetic distance measures.

**Results:**

PATRISTICv1.0 is a java program that calculates patristic distances from large trees in a range of file formats and allows graphical and statistical interpretation of distance matrices calculated by other programs.

**Conclusion:**

The software overcomes some logistic barriers to analysing signals in sequences. In additional to calculating patristic distances, it provides plots for any combination of matrices, calculates commonly used statistics, allows data such as isolation dates to be entered and reorders matrices with matching species or gene labels. It will be used to analyse rates of mutation and substitutional saturation and the evolution of viruses. It is available at  and requires the Java runtime environment.

## Background

Phylogenetic trees are standardly used to analyse gene families as well as species ancestry [1]. A patristic distance is the sum of the lengths of the branches that link two nodes in a tree, where those nodes are typically terminal nodes that represent extant gene sequences or species. A matrix of patristic distances calculated from a tree for all pairs of genes or species summarizes the genetic change, or phylogenetic change, represented in the tree and the data. The distances may be used to analyse the rate of change and may be compared with other measures of genetic difference, such as the total change (evolutionary distance) or components of change such as those indicated by transitions, transversions, gene rearrangements or recombination [2-4]. In one significant example, the origin of the HIV pandemic was investigated by comparing the patristic distances of dated HIV gene sequences [5]. There are many methods and programs for finding, building, or testing trees but only one is known to us that calculates patristic distances and it does so only from maximum parsimony trees, which by definition only represent a fraction of genetic change [6].

## Implementation

PATRISTICv1.0 is a Java program that can be used as an applet on our website or downloaded. It calculates patristic distances from trees, generates scatter plots from ordered pairs of distances and calculates correlation coefficients and other statistics from distance matrices. It reads trees in variants of the Newick format, including the NEXUS variant used by the package PAUP [6] and the variants used by the programs MEGA [6], PHYLIP [8], CLUSTALX [9] and TREEPUZZLE [10]. An algorithm that traverses the various textual representations of trees [11] was used to calculate the patristic distances, along with code that permitted different tree-text formats to be read, permitted the easy selection of matrices for plotting from a large number of stored matrices and permitted matrices and plots to be displayed. PATRISTICv1.0 runs on Windows, Mac and Linux systems with the Java Runtime Environment. A patristic distance matrix from a tree of 187 gene sequences was calculated in 12 seconds in a PC with an AMD CPU at 2.2 GHz and 256 RAM using the JRE 1.5. PATRISTICv1.0 was tested by calculating patristic distances by hand across several small trees and in every case the results of the program were found to be accurate.

The program also recognises distance matrices calculated by other programs from other components of sequence data, such as evolutionary distances calculated from pair-wise sequence comparisons. It reads distance matrices generated by the programs MEGA, PAUP and PHYLIP. For the current version, these externally generated matrices must be presented as upper-right or lower-left hemi-matrices or a column. Other measures of that can be converted into distances, such as the isolation dates of virus samples, may be entered for each species or gene as real numbers. If sample times are entered directly PATRISTICv1.0 will generate a matrix of time differences between the species or sequences.

The order of sequences or species represented in a tree almost always differs from the order in the original data file from which the tree was found. Hence to plot patristic distances against distances calculated by other methods or patristic distances from two trees, the program automatically reorders matrices with matching sequence or species labels. Matrices may also be edited and reordered within an editing window.

**Figure 1:**
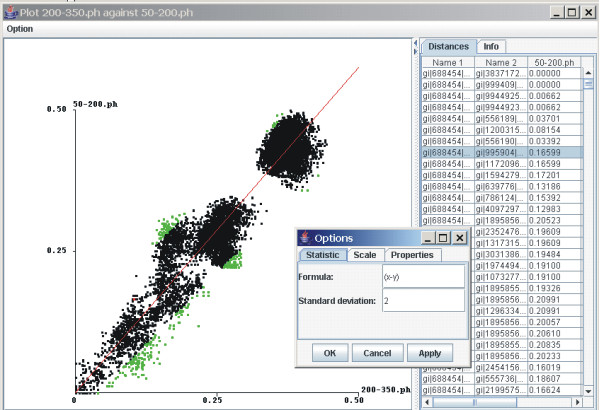
A screenshot of PATRISCTICv1.0 in operation with a plot of patristic distances calculated from two different regions of bunyavirus segment S sequences. The green points represent paired distances that are more than two standard deviations from the mean difference. A regression is shown in red, the distances matrices from which the plot was calculated are shown as columns on the right and an window for entering formulae and altering the scale is shown in the foreground.

A regression is calculated from the ordered pairs of distances when two matrices are plotted against each other (Figure 1) and simple statistics such as the sums of the distances are displayed. Correlation coefficients, differences and quotients between the ordered pairs of distances may also be calculated using PATRISTICv1.0, as may the mean and standard deviation of the differences or quotients, and the program has a facility to enter other formulae so that other statistics can be calculated.

Points on a plot that lie outside a chosen multiple of the standard deviation are identified by colour. Points are also automatically identified on a plot when the mouse cursor is moved over them. A zoom feature allows the user to focus on a specific part of a plot by choosing the minimum and maximum distances for the two axes which correspond to the two distance matrices. Plots of distance matrices may be inverted relative to the axes by a single mouse click. The user can also determine the scale used on the axes as well as the dot size.

Plots may be saved as postscript or jpeg files allowing editing in graphics software in a vectorial or bitmap format. The program also allows distance matrices to be saved in a coma separated value format (CSV) as a full matrix or as columns so that they may be entered into a spreadsheet program. Matrices can also be saved in the DIP format used by the software DIPLOMO [12].

## Results and Discussion

**Figure 2:**
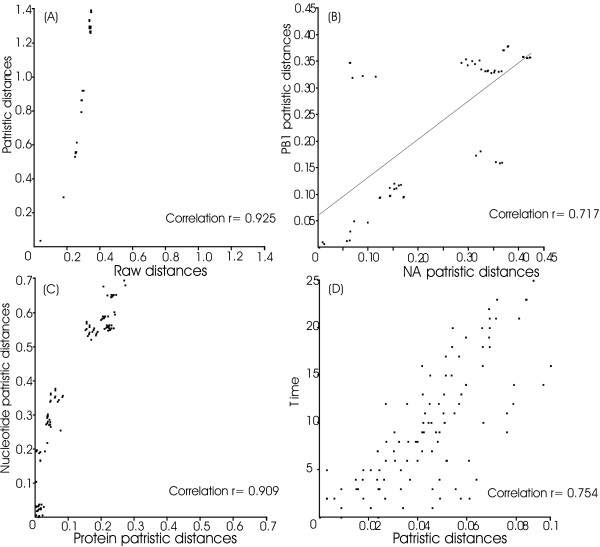
Plots generated using PATRISTICv1.0 of: (A) patristic distances from an ML tree and matching uncorrected evolutionary distances calculated from bunyavirus RNA polymerase gene sequences, (B) patristic distances calculated from the neuraminidase and RNA cap-binding protein genes of a set of influenza A virus N1 subtype isolates with a regression line drawn for all points, (C) patristic distances calculated from the nucleocapsid protein amino acid sequences and the equivalent nucleotide sequences of bunyaviruses, (D) patristic distances from an ML tree of the neuraminidase genes of influenza virus isolates and the differences in years between times of isolation of the isolates.

Maximum likelihood (ML) methods provide the best estimates of evolutionary change and genetic difference by modelling substitutions. Models are used to calculate branch-lengths (genetic change) that take into account the superimposition of substitutions and the similarities between one sequence or taxon and all others represented in the dataset [13]. Hence, some patristic distances from ML trees are greater than the equivalent estimates of genetic distance, when the genetic distances are based on pair-wise comparisons between the raw sequences. The converse also occurs if patristic distances are calculated from a maximum parsimony tree, since characters that do not conform to the cladistic definition of phylogenetic information (autapomorphies and homoplasies) are discounted. To demonstrate one of those effects we used PATRISTICv1.0 to plot patristic distances against uncorrected evolutionary distances, where the patristic distances were calculated from an ML tree of bunyavirus RNA polymerase gene sequences and the evolutionary distances were obtained from pair-wise comparisons of the same sequences without reference to a tree (Figure 2A). It can be seen that whereas the evolutionary (raw) distances increased linearly the patristic distances were greater and increased exponentially.

Another use of patristic distances is to investigate recombinational processes. A low correlation between distances obtained from different genes may indicate a recombinational process such as horizontal gene transfer [14]. Influenza A viruses undergo a recombinational process, known as reassortment, that produces incongruities much like those produced by horizontal gene transfer, but on a smaller scale [15], and the incongruities may be identified as poorly correlated patristic distances. We detected incongruities probably due to reassortment by plotting patristic distances calculated from the neuraminidase (NA) and RNA cap-binding protein (PB2) genes of a set of influenza A virus N1 subtype isolates (Figure 2B); we found that the majority of the points lie close to the regression line and appear to increase linearly, whereas some points fall well off the line. Even when there has been no recombination, different measures of genetic difference from the same set of organisms may not correlate linearly. The rates of change in nucleotide sequences and the amino acid sequences they encode do not vary linearly because of the redundancy in the genetic code and because some sites reach a saturation point of superimposed substitutions. We demonstrated the effect by plotting patristic distances calculated from the nucleocapsid protein amino acid sequences and the equivalent nucleotide sequences of bunyaviruses (Figure 2C); the patristic distances from the nucleotide sequence tree increased at a greater rate than the equivalent patristic distances from the amino acid tree.

Patristic distances have broader uses as it is sometimes of value to compare them with other data, such as data related to time or geographic distribution. For example, comparisons of the times of isolation and the distances between the sequences of influenza A virus strains describe an important process whereby the older strains are being eliminated from global circulation and being replaced by new strains. Figure 2D shows that the patristic distances from an ML tree of the neuraminidase genes of a set of influenza virus isolates correlated with the differences in years between the times of isolation of the isolates.

## Conclusion

A phylogenetic tree usually represents signals drawn from many sites in the sequences from which it was inferred, and it represents a hierarchy of those signals that may be nested. Hence, the relationships between phylogenetic trees and sequences of interest are often complex and obscure. Comparisons of patristic distances and other measures of genetic change allow the relationships to be analysed. PATRISTICv1.0 was developed to permit expedient comparisons and analyses. The program reads trees and distance matrices produced by the most commonly used software without editing. Re-formatting is a time consuming element of multiple sequence and phylogenetic analysis and for that reason PATRISTICv1.0 creates no new intermediate formats. The need to re-order distance data from gene sequences or species was a major barrier that previously hindered analyses and that problem has been solved. The program is likely to be used to analyse recombination, rates of mutation and substitutional saturation and the evolution of rapidly evolving entities such as viruses.

## Availability and requirements

**Project name: **Patristic

**Project home page: **

**Operating system(s): **Platform independent

**Programming language: **Java

**Other requirements: **Java 1.41 or higher 

**Any restrictions to use by non-academics: **None
